# *Lacticaseibacillus rhamnosus* HN001 enhances intestinal barrier function and protects the blood-brain barrier from inflammatory disruption *in vitro*

**DOI:** 10.3389/fphys.2026.1808663

**Published:** 2026-05-08

**Authors:** Dulantha Ulluwishewa, Ajitpal Purba, Vincent Fegan, Narsaa Na, Simon J. O’Carroll, Rachel C. Anderson

**Affiliations:** 1 Bioeconomy Science Institute, Te Rourou, Palmerston North, New Zealand; 2 School of Medical Sciences, The University of Auckland, Auckland, New Zealand

**Keywords:** brain endothelium, gut-brain axis, probiotics, psychobiotics, tight junction

## Abstract

The integrity of the intestinal epithelial and blood-brain barriers is critical for maintaining homeostasis along the gut-brain axis as these interfaces regulate the passage of inflammatory mediators into systemic circulation and their access to the brain. Disruption of these barriers can lead to neuroinflammatory processes implicated in mood disturbances. *Lacticaseibacillus rhamnosus* HN001 has been associated with improved mood outcomes in clinical and preclinical studies, however the mechanistic basis for these effects is unclear. Here, we investigated whether HN001 enhances intestinal and blood-brain barrier function in a two-stage *in vitro* gut-brain axis model. Differentiated Caco-2 epithelial monolayers were co-cultured with HN001 (Stage-1; intestinal epithelial barrier), and the conditioned basal medium from this stage was subsequently applied to hCMEC/D3 endothelial monolayers (Stage-2; blood-brain barrier). HN001 increased transepithelial electrical resistance in Caco-2 monolayers under both aerobic and apical-anaerobic conditions. This was accompanied by transcriptional modulation of tight-junction signaling, including upregulation of *OCLN* and sealing claudins, and downregulation of the pore-forming claudin *CLDN2*. Conditioned basal medium collected from HN001-treated Caco-2 intestinal epithelial monolayers improved barrier resistance in hCMEC/D3 brain endothelial monolayers and mitigated IL-1β-induced barrier disruption. The protective effect was not accompanied by changes in IL-1β-stimulated secretion of IL-6, IL-8, MCP-1, TNF-α or IL-1β, indicating that HN001-derived signals regulate blood-brain barrier function independently of the cytokines measured here. Together, these findings indicate that HN001 supports both the intestinal barrier and the blood-brain barrier integrity within the gut-brain axis. By regulating these barrier interfaces, HN001 may reduce susceptibility to neuroinflammation, suggesting a potential mechanistic basis for its previously reported psychobiotic benefits.

## 1 Introduction

The gut-brain axis is a complex bidirectional communication network linking the gastrointestinal tract and the central nervous system that plays a central role in neurological health and disease (Cryan et al., 2019). Two physical barriers are critical to maintaining homeostasis within this axis: the intestinal epithelial barrier and the blood-brain barrier. The intestinal barrier prevents the translocation of luminal microbes and antigens into the circulation (Camilleri et al., 2012), while the blood-brain barrier regulates the exchange of molecules and immune cells between the blood and the brain (Zlokovic, 2008; Daneman and Prat, 2015). Disruption of the intestinal barrier, often referred to as “leaky gut,” enables microbial products and pro-inflammatory mediators to enter systemic circulation (Bischoff et al., 2014). This systemic inflammation can compromise blood-brain barrier integrity and promote neuroinflammation, which is increasingly recognized as a driver of mood disorders (Varatharaj and Galea, 2017; Pinzi et al., 2025).

Probiotics have emerged as a promising approach to modulate the gut-brain axis through effects on host barrier function, immune activity, and microbial signaling. A subset of probiotics termed “psychobiotics” has been defined as live organisms that, when ingested in adequate amounts, confer mental health benefits to the host (Dinan et al., 2013). Preclinical studies support beneficial effects of probiotics on anxiety- and depression-like behaviors, including reductions in stress-induced corticosterone (Bravo et al., 2011) and alterations in neurotransmitter receptor gene expression in animal models (Dalziel et al., 2023). However, evidence from human trials remains mixed. For example, one meta-analysis of 12 randomized controlled trials reported no significant effect of probiotics on anxiety symptoms (Liu et al., 2018), and a more recent analysis of nine psychobiotic interventions in young people similarly found little evidence of benefit for anxiety (Cohen Kadosh et al., 2021). Likewise, for depression, while some studies report improvements (Wallace and Milev, 2021; Zandifar et al., 2025), others show limited or no effects (Ng et al., 2018; Zagórska et al., 2020). These variable outcomes underscore the need for a deeper understanding of psychobiotic mechanisms of action, particularly their influence on gut and brain barrier integrity.

One strain of particular interest is *Lacticaseibacillus rhamnosus* HN001. Clinical research has shown that maternal supplementation with this strain, originally isolated from dairy cultures, significantly reduced postpartum anxiety and depression scores compared with a placebo (Slykerman et al., 2017). In contrast, a study in undergraduate students found that the same probiotic had no impact on stress, anxiety, or psychological wellbeing (Slykerman et al., 2022). Further complicating the picture, HN001 was associated with beneficial effects on social functioning and anxiety in prediabetic patients on a calorie-restricted diet (Tay et al., 2020). Another recent trial found no overall statistically significant difference in happiness, stress, or anxiety scores between individuals with moderate to high perceived stress in the HN001 and placebo groups (Al Kassaa and Fuad, 2024). However, a *post-hoc* analysis of this trial suggested that in male participants, the probiotic’s impact on happiness was dependent on the intervention’s duration. Supporting the initial positive findings in humans, recent rodent studies demonstrated that HN001 reduces postpartum-anxiety-related behaviors, likely by balancing norepinephrine, dopamine, and serotonin levels (Lonstein et al., 2024). Taken together, these findings highlight that the biological mechanisms underlying the psychobiotic potential of HN001 remain poorly defined.

**Figure 1:**
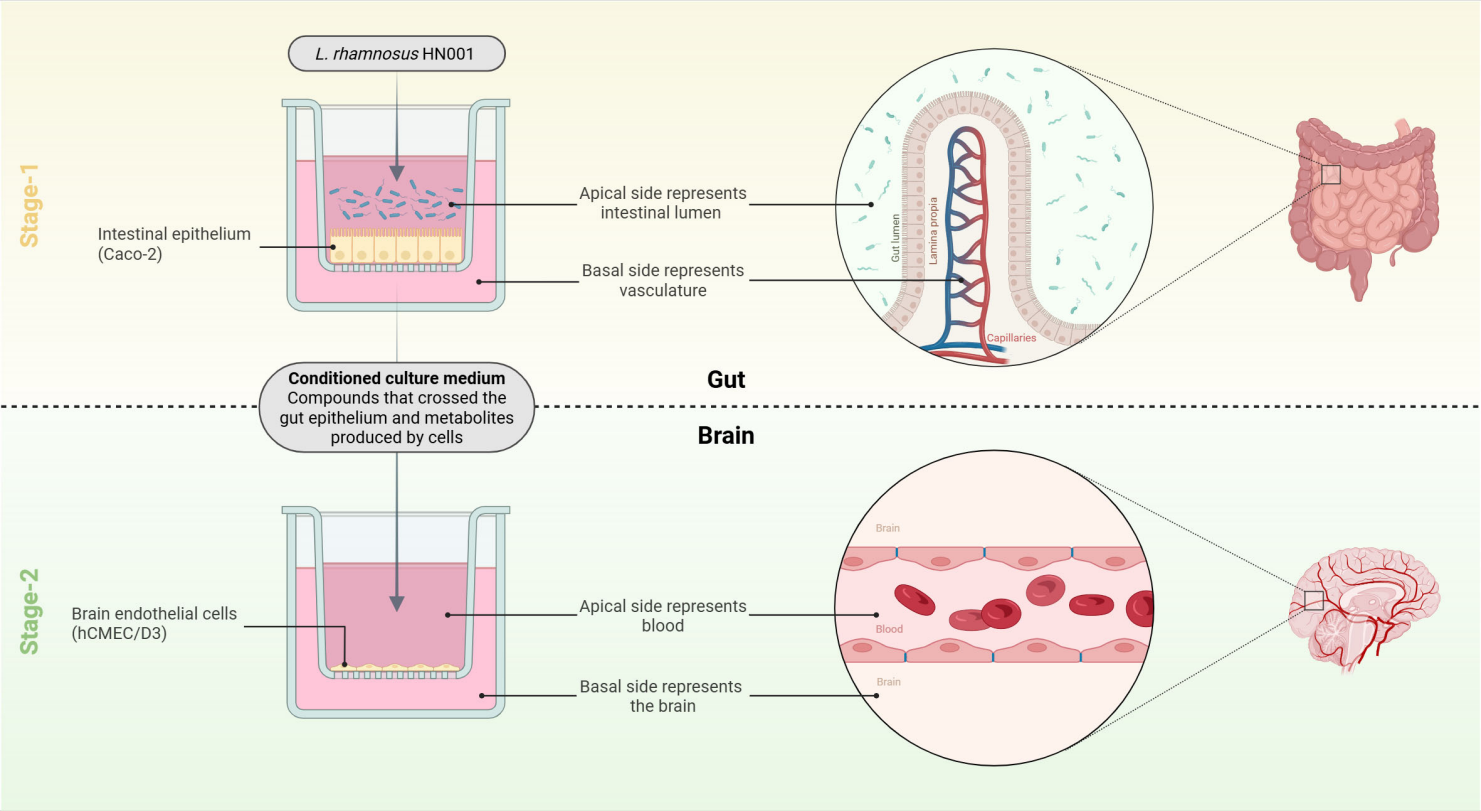
Schematic of the two-stage gut-brain axis model. Stage-1 (‘gut’) represents the intestinal epithelial barrier that separates the lumen from the internal milieu. Following the treatment of the luminal side of the epithelium with bacteria, conditioned culture medium is collected from the basal side. Conditioned media contains compounds that could enter systemic circulation (e.g., bacterial metabolites that crossed the epithelial barrier, and metabolites produced by the epithelium in response to the bacterial treatment). Stage-2 (‘brain’) represents the blood-brain barrier, made up of endothelial cells that regulate passage of molecules between the bloodstream and the brain. Figure created in BioRender. Ulluwishewa, D. (2026) https://BioRender.com/tvzawgs.

Here, we address this knowledge gap by testing the hypothesis that HN001 strengthens intestinal epithelial barrier integrity and, through the release of signaling molecules, enhances blood-brain barrier resistance to inflammatory insults. Specifically, using a two-stage gut-brain axis cell culture model (**Figure 1**), we aimed to (i) investigate the effect of HN001 on intestinal epithelial barrier function *in vitro*, and (ii) assess whether conditioned medium from HN001-treated intestinal cells could influence blood-brain barrier integrity *in vitro* under baseline and inflammatory conditions.

## 2 Materials and methods

### 2.1 Bacterial and mammalian cell culture conditions

*Lacticaseibacillus rhamnosus* HN001 was provided by the Fonterra Research and Development Centre (Palmerston North, New Zealand). Bacterial stocks were stored at -80 °C in De Man-Rogosa-Sharpe (MRS) broth (Merck, Darmstadt, Germany) supplemented with 50% (v/v) glycerol (Sigma-Aldrich, St. Louis, MO, USA). For cultivation, bacteria were grown on MRS agar (Oxoid, Basingstoke, UK) at 37 °C in an anaerobic workstation (Whitley A85 Workstation, Don Whitley Scientific, Bingley, UK) under an atmosphere of 10% CO_2_, 10% H_2_, and 80% N_2_. Agar plates were stored at 4 °C for up to three weeks. Liquid cultures were prepared by inoculating 5 mL of anaerobic MRS broth with a single colony from an agar plate, followed by incubation at 37 °C for 40 hours inside the anaerobic workstation. Anaerobic MRS broth was prepared by placing an open vial of MRS broth inside the anaerobic workstation.

Caco-2 cells (HTB-37) were obtained from the American Type Culture Collection (ATCC) at passage 18 and used for experiments between passages 36 and 39. Cells were maintained in Gibco Medium 199 (M199; Thermo Fisher Scientific, Waltham, MA, USA) supplemented with 10% (v/v) fetal bovine serum (FBS; Moregate BioTech, Hamilton, New Zealand) and 1% (v/v) 100× MEM non-essential amino acids (NEAA; Thermo Fisher Scientific), at 37 °C in a humidified incubator with 5% CO_2_ (Heracell 150i, Thermo Fisher Scientific, Waltham, MA, USA). For co-culture experiments, Caco-2 cells were seeded onto Transwell polyester filter inserts (6.5 mm diameter, 0.4 µm pore size; Corning Inc., Corning, NY, USA) at a density of 8 × 10^4^ cells per insert and cultured for 19 days prior to experimentation.

Human cerebral microvascular endothelial cells (hCMEC/D3) were obtained from Cedarlane Cellutions Biosystems Inc, Ontario, Canada). For experiments cells were cultured between passage P30-P32. Cells were maintained in endothelial cell growth medium consisting of Endothelial Cell Basal Medium-2 (EBM-2; Lonza, AlphaTech, Switzerland) supplemented with 10% (v/v) heat inactivated fetal bovine serum (hiFBS; Moregate BioTech, Hamilton, New Zealand), 0.2 µg/mL hydrocortisone (Sigma Aldrich), 1 µg/mL ascorbic acid (Sigma Aldrich), 10 ng/mL hFGF (Peprotech, 100-18B), 5 ng/mL EGF (Sigma Aldrich), 20 ng/mL IGF-1 (Abcam, Cambridge, UK) and 0.5 ng/mL VEGF (Novus, Centennial, CO, USA) in collagen I coated T75 flasks at 37 °C in a humidified incubator with 5% CO_2_. For Electric Cell-substrate Impedance Sensing (ECIS) experiments, cells were plated onto L-cysteine and collagen I coated 96W20idf plates (Applied Biophysics Inc., Troy, NY, USA). For investigating the cytokine response, cells were plated on collagen-coated polyester filter inserts (6.5 mm diameter, 0.4 µm pore size; Corning Inc., Corning, NY, USA) at a density of 2 × 10^4^ cells per insert and cultured for 5 days prior to experimentation in EBM-2 containing EGM-2 SingleQuots^®^ (2% (v/v) FBS, 0.2 µg/mL hydrocortisone, 75 µg/mL ascorbic acid, 10 ng/mL hFGF-1, 5 ng/mL R3-IGF-1, 2 ng/mL VEGF, 30 µg/mL gentamycin, 15 ng/mL amphotericin, and 1 ng/mL heparin; Lonza).

### 2.2 Measurement of intestinal barrier integrity using the TEER assay

The effect of *L. rhamnosus* HN001 on intestinal epithelial barrier integrity (Stage-1) was assessed by measuring transepithelial electrical resistance (TEER) under both aerobic and apical anaerobic conditions. The protocol for TEER measurements in aerobic conditions using the cellZscope system (nanoAnalytics GmbH, Münster, Germany) has been described in detail by Purba et al. (2025). For TEER measurements under apical anaerobic conditions, a proprietary dual-environment co-culture (DECC) system was employed (Ulluwishewa et al., 2015; Maier et al., 2018). This system allows differentiated Caco-2 cells grown on Transwell inserts to be exposed to an anaerobic environment on the apical side while maintaining viability via oxygen diffusion through the semi-permeable membrane from the aerobic basal compartment. The method was applied as detailed in Zhang et al. (2024).

Briefly, on the day prior to co-culture experiments, the resistance of each Transwell insert containing differentiated Caco-2 monolayers was measured using an EndOhm-6G chamber and EVOM2 meter (World Precision Instruments). After ensuring the TEER was above 400 Ω·cm², the apical medium was replaced with 260 µL of M199 supplemented with 1% (v/v) NEAA. On the day of the experiment, 800 µL of cell culture medium was added to each well of the cellZscope, and 3 mL to each well of the DECC system. Transwell inserts were then placed into the respective wells. The DECC system was transferred into the anaerobic workstation, and Caco-2 monolayers were incubated under apical anaerobic conditions for approximately one hour before co-culture with bacteria.

Forty-hour old bacterial cultures (40 µL) were used to inoculate 5 mL of anaerobic MRS broth and grown for 16 hours under anaerobic conditions to reach stationary phase. Cultures were then centrifuged at 2,500 × *g* for 10 min (Eppendorf A-4–81 rotor, Eppendorf 5810 R centrifuge, Hamburg, Germany), and the resulting pellets were resuspended in anaerobic cell culture medium (without FBS) within the anaerobic workstation. Bacterial concentrations were estimated using a Petroff-Hausser counting chamber (Hausser Scientific, Horsham, PA, USA). For co-culture, the apical medium in the Transwell inserts was replaced with 260 µL of anaerobic cell culture medium containing 1.4 × 10_8_ bacteria/mL. This resulted in a multiplicity of infection of 1000 which is equivalent to the previously chosen effect dose of OD_600nm_ 0.9 (Anderson et al., 2010). For untreated controls, the medium was replaced with an equal volume of anaerobic medium without bacteria.

TEER measurements were taken using electrodes integrated into the co-culture chamber and connected to a cellZscope controller and software (v4.3.1; nanoAnalytics). Baseline TEER was recorded immediately prior to treatment. TEER values were then measured hourly for 12 hours. The percentage change in TEER was calculated using the following formula:


$$\text{Change in TEER }\left(\%\right)=\left[\left(\text{TEE}{\text{R}}_{t}/\text{TEE}{\text{R}}_{0}\right)\;\times \;100\right]\;-\;100$$

where TEER_t_ is the resistance at a given timepoint, and TEER_0_ is the baseline resistance.

Only monolayers with TEER values >400 Ω·cm² at both baseline and 1-hour post-treatment were included in statistical analysis, as values ≤400 Ω·cm² indicate compromised barrier integrity. Four independent assays were conducted, resulting in a total of 11–18 biological replicates per treatment group.

### 2.3 Collection of cell lysate and conditioned basal medium from the Caco-2 cell model

The cell lysate and conditioned basal medium were collected from co-cultures of Caco-2 cells and *L. rhamnosus* HN001 to assess its effect on tight junction gene expression and brain endothelial barrier integrity (Stage-2), respectively. The co-culture setup was conducted similarly to the TEER assay described in Section 2.2 using Transwells in 24-well plates in aerobic cell culture conditions (37 °C in a humidified incubator with 5% CO_2_). The experiment was conducted in three independent runs, with four biological replicates per treatment group, for a total of n=12 per treatment. On the day prior to the experiment, the resistance of each Transwell insert containing differentiated Caco-2 monolayers was measured to ensure TEER was above 400 Ω·cm², and the apical medium was replaced with M199 supplemented with 1% (v/v) NEAA (without FBS). On the day of the experiment, the apical medium in the Transwell inserts was replaced with 260 µL of anaerobic cell culture medium containing 1.4×10^8^ bacteria/mL (multiplicity of infection of 1000) for the treatment group, or an equal volume of anaerobic medium without bacteria for the untreated control. Following an 8-hour co-culture period, the cell culture medium was removed from the Transwell insert, and 200 µL of RNAprotect Cell Reagent (Qiagen, Hilden, Germany) was added to stabilize the RNA and detach the cells. The resulting cell lysate was stored at −80 °C until analysis. In addition, the basal medium from each well was collected and immediately stored at -80 °C until further analysis.

### 2.4 Measurement of intestinal cell tight junction gene expression using NanoString

Total RNA was extracted from the stabilized cell lysate using an RNeasy mini kit (Qiagen) according to the manufacturer’s protocol. The quantity and purity of the extracted RNA were determined using a Nanodrop ND-1000 spectrophotometer (Nanodrop Technologies, Wilmington, DE, USA). Approximately 1000 ng of RNA was used to determine the absolute counts of 90 human tight junction-related genes and 6 reference genes (nCounter Plexset Preselected Human Tight Junction Pathway Panel) using NanoString nCounter technology. This method utilizes molecular barcodes on gene-sequence-specific probes and single-molecule imaging to count RNA copies (Geiss et al., 2008). RNA samples were hybridized by adding 13.5 µL of master mix and 1.5 µL of RNA per tube of a 12-tube strip. The strip was then placed at 67 °C for 22 hours. After hybridization, samples were transferred to the nCounter Prep Station for purification and immobilization in a glass cartridge. The glass cartridge was then transferred to the nCounter Digital Analyzer for data acquisition, which counted and tabulated color codes for each target molecule. Raw data were retrieved from the Analyzer as Reporter Code Count (RCC) files.

For data analysis, RCC files were imported into nSolver Analysis Software (version 4.0.70; NanoString Technologies Inc., Seattle, WA, USA). Samples underwent the software’s quality control routine with the following criteria: (1) imaging: fields of view registration <75%; (2) binding density outside the 0.05 to 2.25 range; (3) positive control linearity: positive control R² value <0.95; and (4) positive control limit of detection: 0.5 fM positive control ≤2 standard deviations above the mean of the negative controls. All samples used for statistical analysis passed the quality control routine. After quality assurance, the data were sequentially normalized to the geometric mean of the respective internal positive controls and the following reference genes: *ABCF1*, *GUSB*, *HPRT1*, *LDHA*, *POLR1B*, and *RPLP0*. Genes with low expression were excluded from statistical analysis. The cut-off threshold was set as the mean plus two standard deviations (SD) of the negative control counts. Genes for which more than half of the samples fell below this threshold were excluded.

### 2.5 Measurement of blood-brain barrier integrity using the ECIS assay

The effect of Caco-2 basal conditioned media on brain endothelial barrier resistance, a core component of the blood-brain barrier, was assessed by measuring the electrical resistance of hCMEC/d3 monolayers using an ECIS ZƟ reader (Applied Biophysics Inc., Troy, NY, USA). ECIS measures the impedance of an alternating current passed across a cell monolayer, which is directly proportional to the integrity of the cell barrier. Conditioned basal media (“HN001 stream” and “control stream”) was collected as described in Section 2.3.

Ninety-six-well culture plates (96W20idf, Applied Biophysics Inc.) were coated with 200 µL of a 1.21 mg/mL L-cysteine solution for 15 minutes, followed by a 50 µL coating of a 150 µg/mL collagen I solution for one hour, prior to cell seeding. The wells were washed twice with sterile water between coating steps. The experiments were conducted in two ways: unchallenged cells treated with conditioned basal media from the Stage-1 intestinal barrier experiments, and challenged cells pre-treated with the same conditioned basal media before an inflammatory insult.

For the unchallenged barrier integrity assessment hCMEC/D3 cells (passage number P30-P32) were seeded at a density of 2×10^4^ cells per well in 200 µL of media. The plates were placed onto an ECIS ZƟ reader inside a cell culture incubator (37 ˚C, 5% CO_2_) immediately after seeding, and the electrical resistance of the cell monolayers was continuously monitored. After 48 hours of incubation, the media in the wells were aspirated and replaced with 100 µL of fresh media. Once the cell monolayer had stabilized at approximately 72 hours, 100 µL of conditioned basal medium from the Caco-2 experiments was added to the wells. The effect of the treatment was monitored between 72–139 hours using the ECIS software (Applied BioPhysics) and a normalized barrier resistance (Rb) value was obtained as a measure of paracellular junctional integrity (Robilliard et al., 2018). For the cytokine challenge hCMEC/D3 barrier integrity assessment, cells were plated and placed on the ECIS ZƟ reader as above. A media change and pre-treatment with conditioned media was conducted at the 48-hour timepoint. The cells were pre-treated by replacing the media in the wells with 195 µL of a 1:1 mixture of conditioned basal Caco-2 medium and endothelial growth medium. At the 72-hour timepoint, a mild inflammatory challenge was initiated by adding 5 µL of a 4 ng/mL interleukin-1β (IL-1β) solution to each well, resulting in a final IL-1β concentration of 0.1 ng/mL. ECIS measurements were carried out between 72–119 hours as above.

### 2.6 Measurement of blood-brain barrier cytokine response

The effect of Caco-2 conditioned basal medium on brain endothelial cytokine responses was assessed using hCMEC/D3 monolayers cultured on Transwell inserts. Conditioned basal media (“HN001 stream” and “control stream”) was collected as described in Section 2.3. The brain endothelial cytokine response assay was performed in two independent runs. In each case, conditioned medium from a single Caco-2 run was used directly in a corresponding hCMEC/D3 run (n = 4 per treatment per run). Conditioned medium was stored at -80 °C until used.

Endothelial monolayers (5 days post-seeding) were pre-treated by replacing the apical medium with a 1:1 mixture of EGM-2 and conditioned medium or, for controls, endothelial growth medium and M199 supplemented with NEAA and FBS (200 µL per insert). The basal compartment was supplied with 800 µL of fresh endothelial growth medium. Plates were incubated for 24 hours at 37 °C in a humidified 5% CO_2_ atmosphere. Following pre-treatment, inserts were challenged with IL-1β by adding 20 µL of IL-1β stock solution (1.1 ng/mL in endothelial growth medium) to achieve a final concentration of 0.1 ng/mL. Unchallenged controls were spiked with 20 µL of endothelial growth medium alone. Cells were incubated for a further 42 hours under identical conditions. At the end of the incubation period, basal culture medium was collected and stored immediately at -80 °C until analysis.

Cytokine concentrations (IL-6, IL-8, MCP-1, TNFα, and IL-1β) in the Stage-2 conditioned basal media were quantified using the MILLIPLEX^®^ Human Adipocyte Magnetic Bead Panel (HADCYMAG-61K, Merck, Rahway, NJ, USA), following the manufacturer’s instructions. Assays were performed by the Massey Nutrition Laboratory using the Bioplex 200 System (Luminex, Belgium), and all samples were analyzed in duplicate.

### 2.7 Statistical analysis

Statistical analysis was performed using R version 4.3.0 (R Core Team, 2023), with plots generated using the ggplot2 package (Wickham, 2016).

For the TEER and ECIS data, the effect of treatment on the change in resistance over time was compared using a linear mixed-effects model to account for repeated measurements on the same monolayers. For TEER data, models were fitted using the restricted maximum-likelihood (REML) method from the nlme package (Pinheiro et al., 2024). The statistical model included the effects of treatment, time, and their interaction as fixed effects. Transwell inserts nested within experimental runs (considered as blocks) were included as a random effect. For the ECIS data, models were fitted using the lme4 package (Bates et al., 2015). The models included the effects of time, treatment, and their interaction as fixed effects. The well nested within a block, as well as the replicate (where a sample from a given well from the Caco-2 model was considered a single replicate), were included as random effects. The replicate factor was omitted from the model used to analyze data from the challenged blood-brain barrier model, as it did not explain any variability in that dataset. If the treatment × time interaction was significant (P < 0.05), pairwise comparisons were performed on the estimated marginal means using the emmeans package (Lenth, 2024). The false discovery rate (q) was applied to adjust for multiple comparisons, with differences considered statistically significant when q < 0.05.

Multivariate analysis of scaled gene expression data was conducted using a multilevel principal component analysis (PCA) with the mixOmics R package version 6.24.0 (Rohart et al., 2017). The multilevel approach was utilized to account for the variance associated with the different nanostring runs. For each individual gene, permutation ANOVAs were performed on the normalized counts using the lmPerm package version 2.1.0 (Wheeler and Torchiano, 2016). The permutation models tested the effect of the treatment while controlling for the independent experimental runs and nanostring runs as blocking factors. Fold changes for each treatment group were calculated relative to the control group to correspond with the permutation ANOVA P-values. This data was then mapped to the Kyoto Encyclopedia of Genes and Genomes (KEGG) pathways database (Kanehisa and Goto, 2000) using the KEGG mapping tools (Kanehisa et al., 2022).

Cytokine data were analyzed using linear mixed-effects models with the lme4 package. To meet model assumptions, cytokine concentrations were log-transformed prior to analysis. For each cytokine, treatment was included as a fixed effect and experiment as a random intercept to account for variation between independent experimental runs. ANOVA was used to assess the main effect of treatment, and pairwise comparisons between treatments were performed on estimated marginal means using the emmeans package with Tukey’s *post-hoc* adjustment for multiple testing, and significance was accepted at P < 0.05.

## 3 Results

### 3.1 HN001 improves intestinal epithelial barrier function under aerobic and apical anaerobic conditions

**Figure 2:**
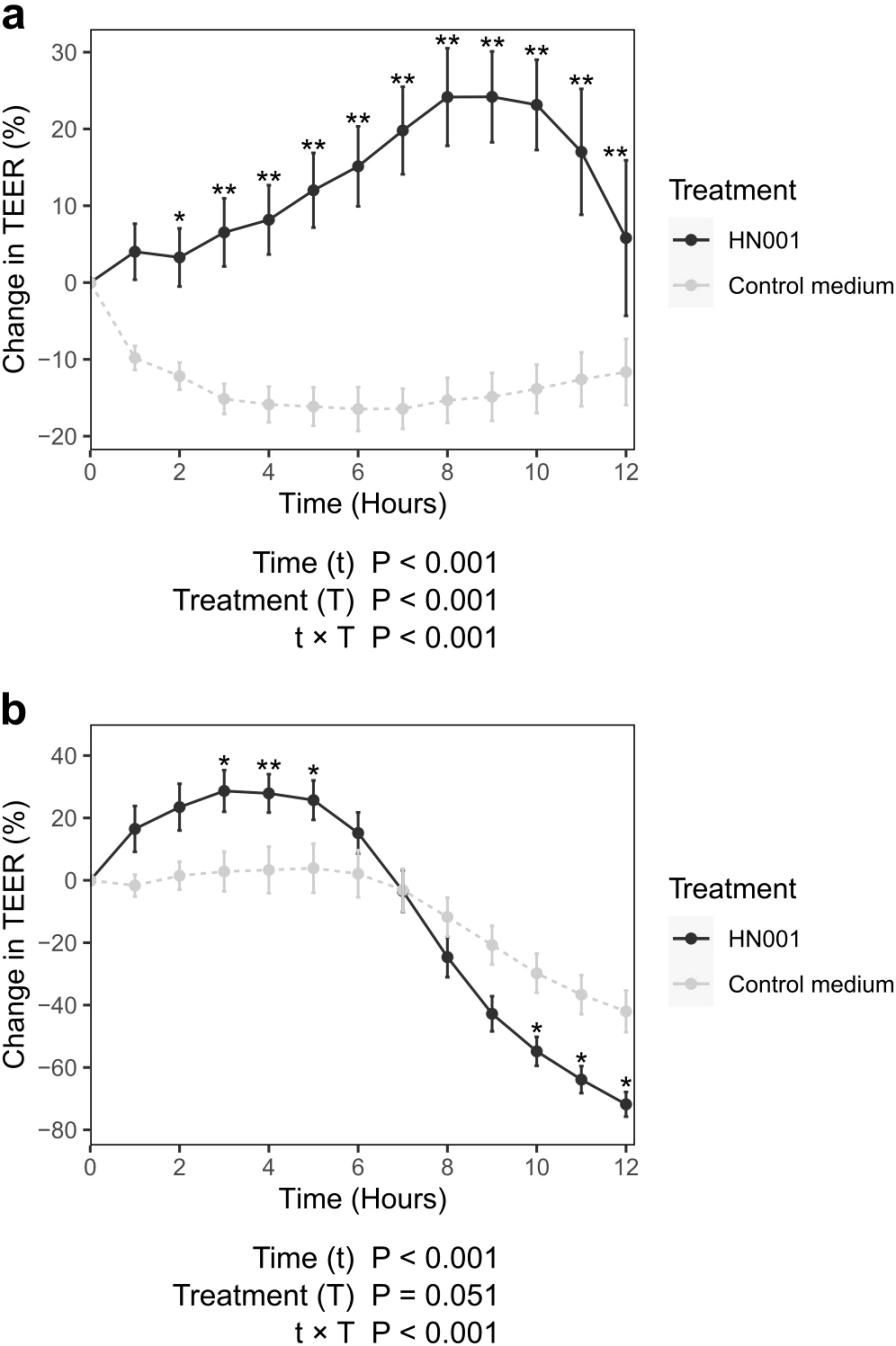
Effect of probiotic on gut epithelial barrier function. Twenty-day old Caco-2 monolayers were co-cultured with *Lacticaseibacillus rhamnosus* HN001with a multiplicity of infection of 1000, or cultured without probiotic bacteria (control medium). Graphs show the mean (± SEM) change in TEER. Experiments were carried out under **(a)** conventional (n = 12-18; 3 experiments, 3–10 samples per treatment per experiment), and **(b)** apical-anaerobic conditions (n = 11-16; 4 experiments, 2–6 samples per treatment per experiment). P-values from ANOVA for effects of time (t), treatment (T), and their interaction (t × T) are shown below each graph. *q < 0.05, **q < 0.01 compared to no treatment control at the given timepoint.

We investigated the effect of *L. rhamnosus* HN001 on intestinal epithelial barrier function *in vitro* by measuring TEER as an indicator of intestinal epithelial cell tight junction integrity under both aerobic and apical anaerobic conditions over time. For the Caco-2 cell monolayers treated with control medium, the TEER was stable for longer in aerobic conditions than apical anaerobic conditions. In aerobic conditions, initially the TEER dropped by estimated marginal mean (emmean) ± standard error (SE) of 9.81 ± 2.42% (P < 0.001), after the media change but then it was consistent from 3 to 12 hours (**Figure 2a**). Conversely, under apical anaerobic conditions, the TEER was stable for 8 hours and then decreased from 9 hours onwards (**Figure 2b**), likely due to a reduction in oxygen in the basal medium. However, all monolayers retained a TEER >400 Ω·cm² which indicates that the barrier function was still intact.

For the Caco-2 cell monolayers treated with HN001 the TEER was significantly higher compared to those treated with control medium, which indicates that HN001 improved the barrier integrity. This period of enhanced TEER was longer under aerobic conditions (2–12 hours post-addition of HN001; **Figure 2a**) compared to apical anaerobic conditions (3–5 hours post addition of HN001; **Figure 2b**). In both cases, the TEER decreased after prolonged treatment with HN001 likely due to the decrease in pH caused by the production of lactic acid by the bacterium which was apparent by the change in color of the medium which contained the pH indictor phenol red. This drop in pH (as evident by a change in media color) occurred earlier in apical anaerobic conditions, which suggests that more lactic acid was produced in anaerobic conditions compared to aerobic conditions.

### 3.2 HN001 modulates tight-junction-related gene expression in intestinal epithelial monolayers

**Figure 3:**
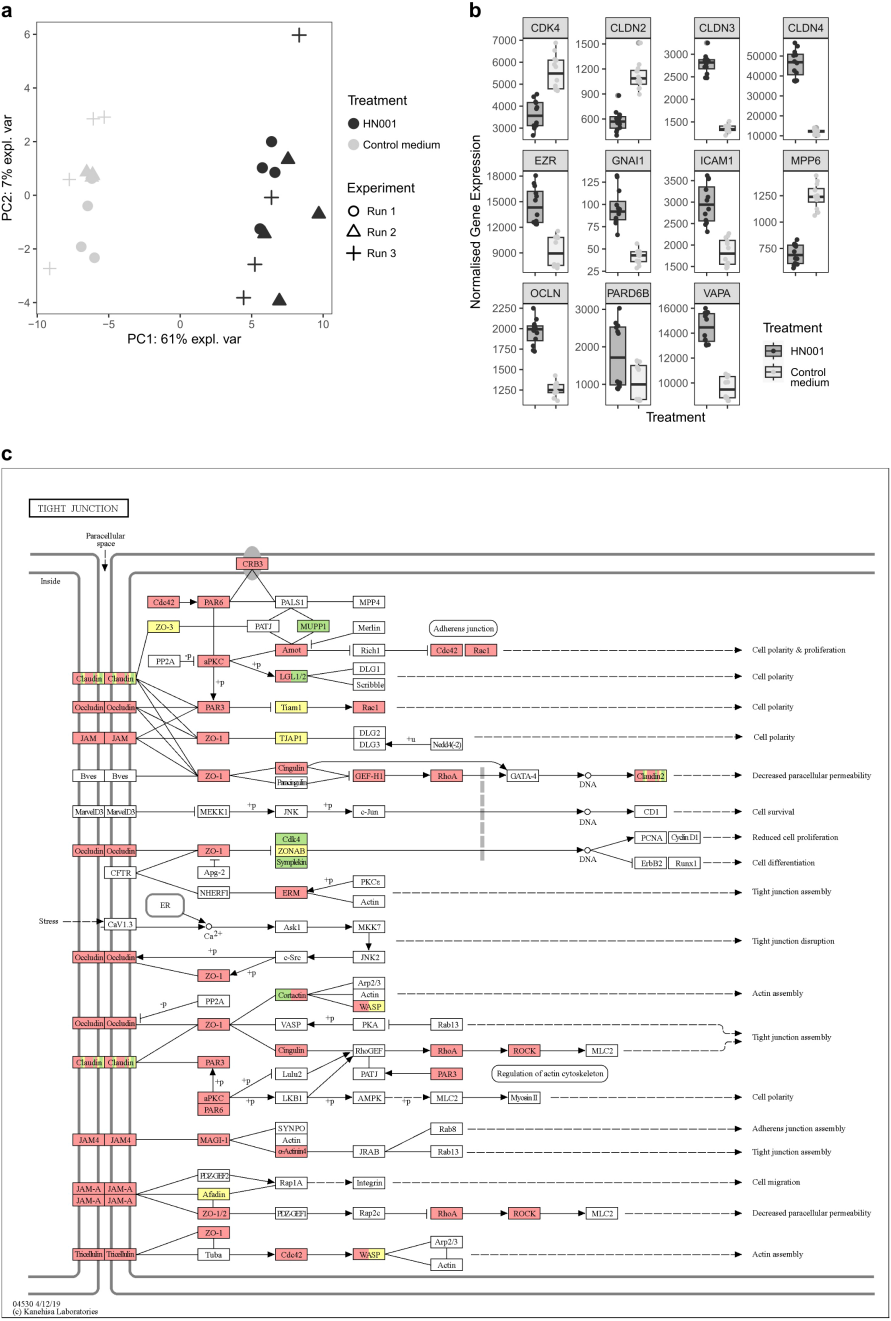
Effect of probiotic on gut epithelial gene expression. **(a)** Principal component analysis (PCA) plot of gene expression profiles of Caco-2 monolayers co-cultured with *Lacticaseibacillus rhamnosus* HN001 and untreated Caco-2 monolayers (Control medium) for 8 hours in aerobic conditions (n = 12; 3 experiments, 4 samples per treatment per experiment). The plot shows the first two principal components, which account for 61% and 7% of the total variance, respectively. Different symbols indicate independent experimental runs. **(b)** Box and whisker plots of differentially expressed genes (P < 0.05) with a fold change of >1.5 or <-1.5. The plots indicate the median (middle line), first and third quartile (boundaries of box), and 1.5 times the interquartile range (whiskers). **(c)** Transcriptional changes projected onto the Kyoto Encyclopedia of Genes and Genomes (KEGG) tight junction pathway. Genes with significantly increased expression in Caco-2 cells co-cultured with *Lacticaseibacillus rhamnosus* HN001 (compared to untreated Caco-2 cells) are depicted in red (P < 0.05), genes with decreased expression are green (P < 0.05), and genes with no significant change are yellow (P > 0.05).

Based on the results of the TEER assay, we chose to investigate the effect of HN001 on tight junction-related gene expression under aerobic conditions. Under apical anaerobic conditions the pH dropped more quickly, likely due to the facultative anaerobic bacterium being more metabolically active. This build-up of acid over time and subsequent drop in TEER was an artifact of the closed model system and is not likely to occur under the dynamic conditions in the gut. For the gene expression analysis, we chose the timepoint of 8 hours of co-culture where the increase in TEER compared to control cells was at its greatest in aerobic conditions. Of the 90 tight-junction-related genes assessed, 10 were excluded from analysis because their expression levels fell below the detection threshold (22.45) in more than half of the samples. The remaining 80 genes were retained for statistical analysis. Principal component analysis (PCA) revealed clear separation of samples according to treatment, with HN001 co-cultured cells clustering distinctly from untreated control monolayers along the first principal component (PC1, 61% of explained variance; **Figure 3a**). Importantly, samples from the three independent runs overlapped within their respective treatment groups, indicating that the transcriptional response to HN001 was consistent across experiments.

Examination of individual transcripts confirmed that HN001 broadly altered the expression of tight-junction-related genes in Caco-2 monolayers (**Supplementary Table 1**). HN001 upregulated 55 genes and downregulated 12 genes, while no change (P > 0.05) was observed for 13 genes. Expression was altered for genes encoding junctional components, cytoskeletal regulators, and polarity proteins, indicating a wide-ranging impact of HN001 on epithelial barrier pathways. To focus on the most pronounced responses, we examined genes with fold changes greater than 1.5 (**Figure 3b**). An increase in expression levels was observed for CLDN3, CLDN4, EZR, OCLN, ICAM1, and GNAI1, while decreases in expression were seen for CLDN2, MPP6, and CDK4. These changes suggest that HN001 enhances expression of transcripts supporting junction assembly and cytoskeletal organization, while downregulating genes linked to increased permeability or cell proliferation.

Projection of these transcriptional changes onto the KEGG tight junction pathway emphasized their functional distribution (**Figure 3c**). Upregulated genes mapped broadly across barrier-forming and cytoskeletal nodes, whereas downregulated transcripts clustered within subsets of claudins and polarity regulators. Together, these findings demonstrate that *L. rhamnosus* HN001 induces a consistent and coordinated remodeling of tight junction-related gene expression in intestinal epithelial cells, in a manner consistent with the corresponding functional TEER data.

### 3.3 Conditioned basal medium from HN001-treated Caco-2 monolayers improves healthy barrier function and mitigates IL-1β-induced loss-of barrier function in brain endothelial cells

**Figure 4:**
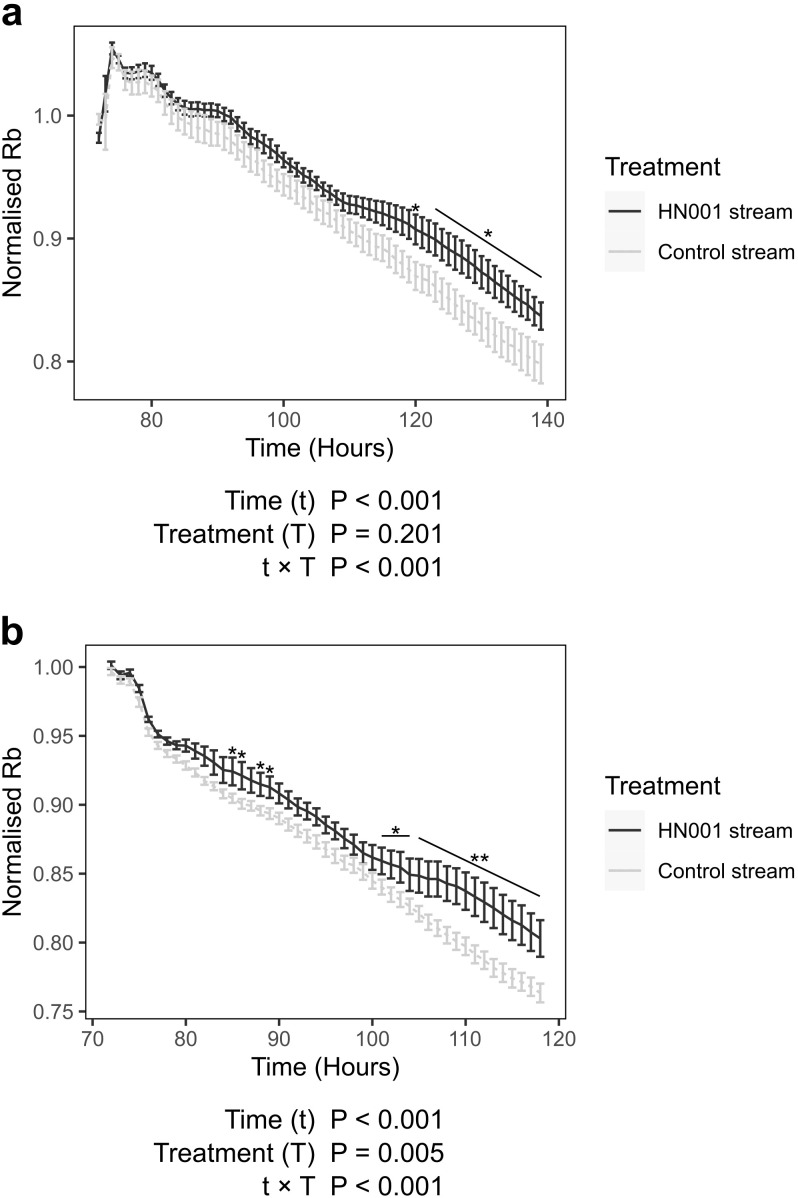
Effect of Stage-1 conditioned basal medium on blood-brain barrier function. Conditioned basal medium was collected from Caco-2 monolayers either co-cultured with *Lactobacillus rhamnosus* HN001 (HN001 stream) or cultured without bacteria (Control stream), and applied to hCMEC/D3 cells prior to measurement of barrier resistance using Electric Cell-substrate Impedance Sensing (ECIS). Graphs show the mean (+/- SEM) normalized barrier resistance (Rb). Cells were **(a)** treated at 72 hours post-seeding (n = 7-12; 2 experiments, 3–8 samples per treatment per experiment), or **(b)** pre-treated at 48 hours post-seeding and challenged with IL-1β 24 hours later (n = 9-12; 3 experiments, 2–5 samples per treatment per experiment). P-values from ANOVA for effects of time (t), treatment (T), and their interaction (t × T) are shown below each graph. *q < 0.05, **q < 0.01 compared to control stream.

We next assessed the effect of conditioned basal medium from Stage-1 Caco-2 cultures on blood-brain barrier function, using hCMEC/D3 monolayers as an *in vitro* model. Barrier integrity was measured by ECIS and expressed as normalized resistance (Rb). When treated at 72-hours post-seeding, hCMEC/D3 monolayers exposed to the HN001 stream (conditioned medium from HN001-treated Caco-2 monolayers in Stage-1) displayed significantly higher Rb values than those treated with the control stream, with differences evident at 120 hours and persisting from 123 to 139 hours post-seeding (**Figure 4a**). These data indicate that the HN001 stream enhanced brain endothelial barrier function.

When cells were pre-treated with the control stream at 48 hours post-seeding and subsequently challenged with IL-1β 24 hours later, the normalized Rb dropped by an estimated marginal mean (emmean) ± standard error (SE) of 0.231 ± 0.006 (P < 0.001) over the following 46 hours (**Figure 4b**), confirming that IL-1β disrupted blood-brain barrier integrity. While barrier disruption was also observed in cells pre-treated with the HN001 stream (drop of 0.193 ± 0.007; P < 0.001), Rb values remained significantly higher compared with cells pre-treated with the control stream. Thus, the HN001 stream was able to partially mitigate IL-1β induced blood-brain barrier dysfunction.

### 3.4 Conditioned basal medium from HN001-treated Caco-2 monolayers does not alter cytokine responses in hCMEC/D3 monolayers.

**Figure 5:**
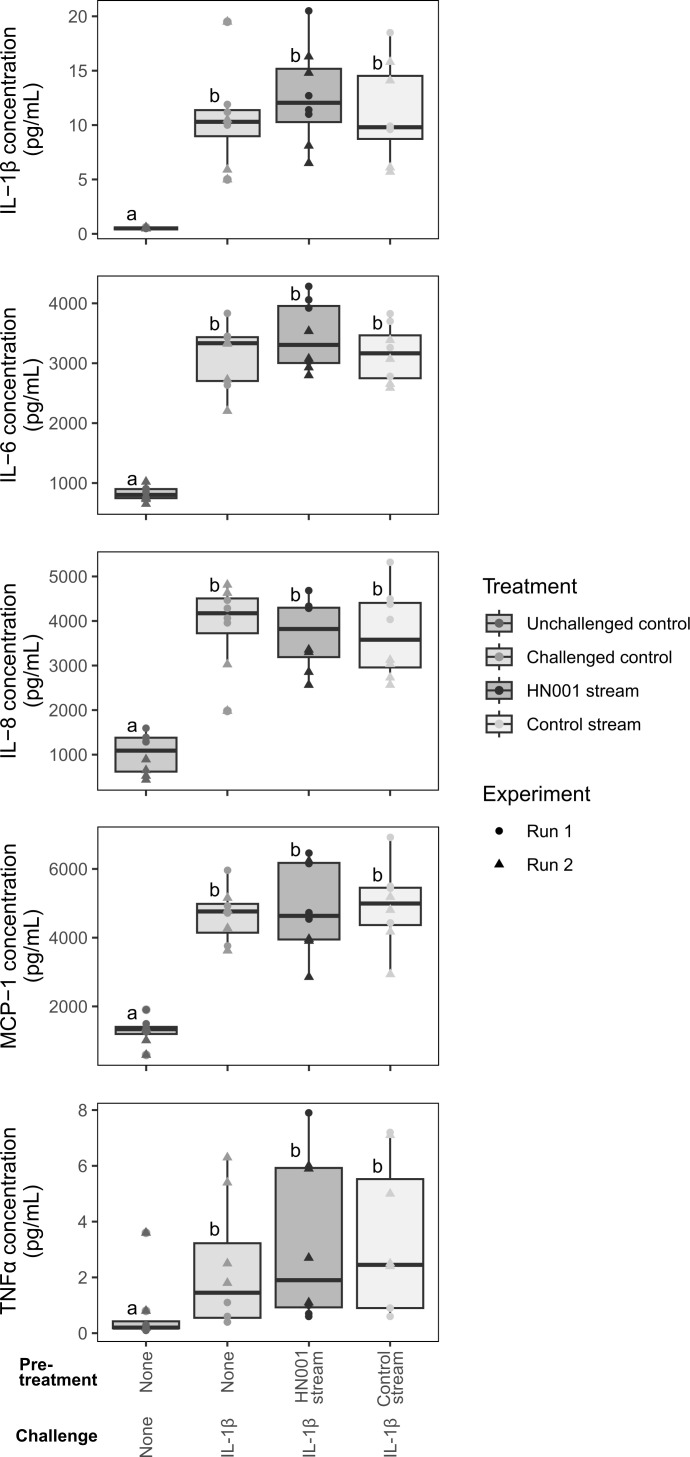
Effect of conditioned basal medium from Caco-2 monolayers on cytokine release in hCMEC/D3 cells. Five-day old hCMEC/D3 monolayers were apically pre-treated with either the HN001 stream (conditioned basal medium from *Lacticaseibacillus rhamnosus* HN001-treated Caco-2 monolayers), the control stream (conditioned basal medium from untreated Caco-2 monolayers), or fresh medium, followed by stimulation with IL-1β (0.1 ng/mL) for 42 hours. Cytokine concentrations in the basal compartment were quantified by multiplex bead assay. Box and whisker plots indicate the median (middle line), first and third quartile (boundaries of box), and 1.5 times the interquartile range (whiskers) of IL-1β, IL-6, IL-8, MCP-1, TNF-α. Symbols represent data points from two independent experimental runs, with n = 4 per treatment per run. Treatments which do not share the same letter (a, b) are significantly different (P < 0.05).

We next investigated whether conditioned basal medium from Stage-1 Caco-2 cultures influenced cytokine release from hCMEC/D3 monolayers during a 42-hour inflammatory challenge (**Figure 5**). In the absence of IL-1β challenge, basal cytokine concentrations (IL-1β, IL-6, IL-8, MCP-1, and TNF-α) were low across all groups. Following apical IL-1β challenge, basal cytokine concentrations increased markedly, confirming that the hCMEC/D3 model mounted a pro-inflammatory response.

Pre-treatment with either the HN001 stream or the control stream for 24 hours did not significantly modify the IL-1β-induced increases in cytokine secretion. Across both independent runs, concentrations of IL-1β, IL-6, IL-8, MCP-1, and TNF-α in the basal compartment were similar between HN001 stream-pretreated cells and control stream-pretreated cells. These findings suggest that the protective effect of the HN001 stream on blood-brain barrier integrity (**Figure 4**) is unlikely to be mediated through suppression of endothelial release of the measured cytokines, but may involve other barrier-modulating mechanisms.

## 4 Discussion

Our study demonstrates that *L. rhamnosus* HN001 enhances intestinal epithelial barrier integrity and protects the blood-brain barrier from inflammatory disruption *in vitro*. Specifically, HN001 increased TEER values across intestinal epithelial cell monolayers under both aerobic and apical-anaerobic conditions, consistent with transcriptional upregulation of barrier-promoting genes such as CLDN3, CLDN4, and OCLN, alongside downregulation of the pore-forming CLDN2. Furthermore, conditioned medium from HN001-treated intestinal epithelial cells enhanced endothelial barrier function in a blood-brain barrier model and attenuated IL-1β-induced barrier disruption. Together, these findings support our hypothesis that HN001 exerts psychobiotic potential by strengthening gut and blood-brain barrier integrity, thereby reducing susceptibility to neuroinflammatory insults. This work indicates a mechanistic basis as to how HN001 may contribute to improved brain health outcomes observed in some human and animal studies (Slykerman et al., 2017; Lonstein et al., 2024).

These findings build on our previous work showing that certain *Lactobacillus* strains improve intestinal epithelial integrity, as indicated by increased TEER and altered tight junction gene expression (Anderson et al., 2010; Zhang et al., 2024). Notably, in the present study, our data show that HN001 induced a broad transcriptional remodeling of the epithelial tight junction signaling network, including upregulation of sealing claudins (e.g., *CLDN3* and *CLDN4*), and downregulation of the *CLDN2*, which encodes a pore-forming claudin that increases paracellular ion flux (Camilleri et al., 2012). The upregulation of *ICAM1*, which encodes a cell-surface adhesion protein involved in epithelial signaling and leukocyte interaction, likely reflects a regulated epithelial response (Bui et al., 2020). In gut epithelia, ICAM1 has been shown to be upregulated by NF-κB signaling and has been linked to epithelial activation and host-microbe communication (Huang et al., 1996; Jobin et al., 1998). In the present study, this upregulation occurred alongside increased expression of barrier-associated genes, including CLDN3, CLDN4, OCLN, and EZR, together with decreased expression of CLDN2 which is consistent with strengthened junctional integrity and reduced paracellular permeability (Balda and Matter, 2009). Thus, the ICAM1 response appears to be part of a broader HN001-induced epithelial remodeling program that aligns with the observed increase in TEER and supports enhanced barrier function. The observed barrier-strengthening effect is consistent with the proposed health-promoting actions of psychobiotics, which include modulation of gut barrier integrity (Dinan et al., 2013; Sarkar et al., 2016).

A novel aspect of this study is the demonstration that soluble factors generated during the interaction between HN001 and intestinal epithelial cells enhance brain endothelial barrier resistance, a core component of the blood-brain barrier, under inflammatory challenge. To our knowledge, this is the first report that shows conditioned medium from intestinal epithelial cells co-cultured with HN001 can directly modulate brain endothelial barrier integrity. This finding is relevant because IL-1β, a key mediator of neuroinflammation, is known to directly cause the breakdown of the blood-brain barrier (Blamire et al., 2000). The ability of HN001-conditioned medium to partially mitigate IL-1β-induced blood-brain barrier dysfunction suggests a pathway by which probiotics in the gut may exert protective effects on the central nervous system. While the identity of the active factors remains unknown, they may include bacterial metabolites such as short-chain fatty acids (which can cross the intestinal barrier) or host-derived anti-inflammatory mediators and growth factors induced in Caco-2 cells in response to HN001 (Bischoff et al., 2014; Cryan et al., 2019). Our cytokine assays in hCMEC/D3 cells showed that pre-treatment with the HN001 stream did not attenuate the IL-1β-induced release of IL-6, IL-8, MCP-1, or TNF-α. This suggests that the protective effect of the HN001 stream on blood-brain barrier integrity is not due to the reduced expression of these cytokines, but may involve barrier-modulating pathways, such as altered tight junction dynamics or paracellular signaling.

Our *in vitro* findings are consistent with clinical and preclinical evidence linking *Lacticaseibacillus rhamnosus* HN001 to improved mood outcomes. Of particular relevance is the effectiveness of HN001 on postpartum mood in humans, where HN001 supplementation during pregnancy reduced symptoms of postpartum anxiety and depression (Slykerman et al., 2017), and altered behaviors similarly in rodents (Lonstein et al., 2024). The postpartum period is characterized by pronounced hormonal, immune, and vascular changes (Pawluski et al., 2017), and the resulting inflammatory response is correlated with the onset of perinatal mood disorders (Osborne and Monk, 2013). As discussed in the review by Kendall-Tackett (2007), pro-inflammatory cytokines can induce a constellation of behaviors, such as alterations in mood, fatigue, and social withdrawal, that strongly mirror the core symptoms of depression. The extent to which these inflammatory mediators influence brain function depends on the integrity of the gut and blood-brain barriers (Kelly et al., 2015; Camilleri, 2019). Disruption of these barriers permits translocation of microbial products, toxins, and pro-inflammatory cytokines from the gut lumen into the circulation, initiating systemic inflammation that, in turn, compromises blood-brain barrier integrity and allows inflammatory mediators to enter the brain and activate neuroimmune pathways (Varatharaj and Galea, 2017). By strengthening intestinal barrier integrity, HN001 may limit systemic inflammation, and through soluble factors released from HN001-treated intestinal cells, it may protect the blood-brain barrier from cytokine-induced disruption. Therefore, our results suggest a plausible mechanism by which HN001 exerts its psychobiotic potential to mitigate symptoms of postpartum and other depressive disorders.

While this study advances understanding of the potential psychobiotic properties of HN001, several limitations constrain mechanistic interpretation of the findings. One example is the absence of tight junction protein abundance and localization data. Although the results showed changes in the gene expression of tight junction protein pathway that aligned with improved physiological outcomes (TEER) in the cells, the intermediate step of altered tight junction protein assembly and distribution can only be inferred. In addition, while host cell responses were characterized, the specific bacterial factors responsible were not determined. Therefore, further work could examine whether these effects are specific to live HN001, shared by other *Lacticaseibacillus* strains, or mediated by secreted metabolites. Similarly, this study did not identify the specific metabolites responsible for the observed effects, because doing so is technically challenging in the current system due to surfactants in cell culture media that are incompatible with metabolomics workflows.

The data presented here were generated using an *in vitro* system designed to capture key features of the intestinal and blood-brain barriers. While this reductionist approach allows controlled investigation of mechanistic interactions, it does not fully represent the complexity of the *in vivo* gut-brain axis. Future studies could therefore employ more advanced co-culture models incorporating immune cells, and mucus-producing goblet cells into the gut barrier model (Stage-1), as well as astrocytes and pericytes to the blood-brain barrier model (Stage-2). Such systems would enable a more physiologically relevant assessment of how probiotics influence barrier function under dynamic, multicellular conditions. Extending these studies to a ‘Stage-3’ neuronal model will help determine whether probiotic-induced barrier strengthening translates into improved neural resilience and mental health outcomes.

## 5 Conclusions

This study provides novel evidence that *L. rhamnosus* HN001 strengthens both intestinal and blood-brain barriers *in vitro*. The findings suggest a mechanistic basis for the psychobiotic action of HN001, which we show regulates tight junction gene expression and enhances intestinal barrier integrity. Furthermore, HN001 may act by releasing soluble factors that cross the intestinal barrier or stimulate intestinal epithelial responses that in turn protect the blood-brain barrier from inflammatory challenge. Importantly, this protective effect was not associated with suppression of endothelial release of the cytokines measured, indicating that the mechanism might be related to barrier-modulating pathways. These findings highlight the potential of HN001 as a dietary strategy to support neurological health by modulating key barriers of the gut-brain axis.

## Data Availability

The data that support the findings of this study are available at https://doi.org/10.57935/AGR.32003778.
