# Supplementary material for: Lacticaseibacillus rhamnosus HN001 enhances intestinal barrier function and protects the blood-brain barrier from inflammatory disruption in vitro
Source: Front Physiol. 2026 May 8;17:1808663. doi: 10.3389/fphys.2026.1808663 (PMC13194050; doi:10.3389/fphys.2026.1808663)
Supplement: Supplementary file 1 [file Table1.docx]

Table S1 Normalized gene expression of tight-junction-related genes in Caco-2 monolayers co-cultured with *Lacticaseibacillus rhamnosus* HN001 or maintained in untreated control medium (no bacteria). **Gene expression was quantified using the NanoString nCounter Human Tight Junction Pathway Panel. Values represent mean (± SEM) normalized expression from three independent experiments (n = 12 per treatment). Of the 90 genes measured, 10 with expression below the detection threshold in more than half of samples were excluded, leaving 80 genes for analysis (shown). P-values indicate differences between HN001-treated and control monolayers (* P < 0.05, ** P < 0.01, *** P < 0.001, ns = not significant).**

| Gene | Mean (± SEM) normalized gene expression | | P-value | Significance |
| --- | --- | --- | --- | --- |
|  | HN001 | Control medium |  |  |
| ACTN1 | 4634 (± 149) | 4128 (± 107) | <0.001 | *** |
| ACTN4 | 20033 (± 583) | 18513 (± 639) | <0.001 | *** |
| AFDN | 2099 (± 159) | 2183 (± 181) | 0.131 | ns |
| AMOTL1 | 6301 (± 262) | 5146 (± 237) | <0.001 | *** |
| ARHGEF2 | 1177 (± 33) | 934 (± 35) | <0.001 | *** |
| ASH1L | 1175 (± 45) | 1146 (± 57) | 0.216 | ns |
| CASK | 5771 (± 269) | 6050 (± 221) | 0.005 | ** |
| CD99 | 9026 (± 126) | 7523 (± 117) | <0.001 | *** |
| CDC42 | 24050 (± 768) | 20092 (± 716) | <0.001 | *** |
| CDK4 | 3617 (± 184) | 5534 (± 225) | <0.001 | *** |
| CGN | 2353 (± 96) | 1909 (± 115) | <0.001 | *** |
| CLDN1 | 8911 (± 265) | 9800 (± 312) | <0.001 | *** |
| CLDN10 | 42 (± 3) | 33 (± 1) | 0.002 | ** |
| CLDN11 | 48 (± 2) | 35 (± 2) | <0.001 | *** |
| CLDN12 | 523 (± 11) | 633 (± 11) | <0.001 | *** |
| CLDN14 | 33 (± 1) | 31 (± 2) | 0.262 | ns |
| CLDN15 | 352 (± 7) | 279 (± 9) | <0.001 | *** |
| CLDN16 | 404 (± 10) | 353 (± 14) | <0.001 | *** |
| CLDN17 | 36 (± 2) | 28 (± 1) | 0.002 | ** |
| CLDN19 | 64 (± 3) | 86 (± 5) | 0.001 | ** |
| CLDN2 | 577 (± 37) | 1118 (± 47) | <0.001 | *** |
| CLDN20 | 30 (± 1) | 26 (± 1) | 0.013 | * |
| CLDN3 | 2784 (± 63) | 1354 (± 24) | <0.001 | *** |
| CLDN4 | 46244 (± 1970) | 12142 (± 307) | <0.001 | *** |
| CLDN6 | 115 (± 12) | 85 (± 6) | 0.061 | ns |
| CLDN7 | 10041 (± 340) | 8609 (± 203) | <0.001 | *** |
| CLDN9 | 39 (± 1) | 35 (± 2) | 0.126 | ns |
| CRB1 | 42 (± 1) | 30 (± 1) | <0.001 | *** |
| CRB3 | 4652 (± 159) | 3139 (± 130) | <0.001 | *** |
| CSNK2A1 | 67 (± 3) | 94 (± 3) | <0.001 | *** |
| CSNK2A2 | 343 (± 17) | 288 (± 11) | 0.009 | ** |
| CSNK2B | 10262 (± 469) | 8728 (± 448) | <0.001 | *** |
| CTNNA1 | 19279 (± 582) | 15975 (± 526) | <0.001 | *** |
| CTNNB1 | 6115 (± 218) | 5247 (± 178) | <0.001 | *** |
| CTTN | 9618 (± 403) | 10324 (± 544) | 0.001 | ** |
| EPB41 | 488 (± 20) | 427 (± 17) | <0.001 | *** |
| ESAM | 437 (± 19) | 347 (± 13) | <0.001 | *** |
| EZR | 14574 (± 616) | 9167 (± 516) | <0.001 | *** |
| F11R | 10499 (± 635) | 7351 (± 516) | <0.001 | *** |
| GNAI1 | 96 (± 6) | 41 (± 2) | <0.001 | *** |
| HCLS1 | 39 (± 2) | 29 (± 1) | 0.001 | ** |
| ICAM1 | 2968 (± 133) | 1834 (± 90) | <0.001 | *** |
| ICAM2 | 121 (± 5) | 118 (± 6) | 0.643 | ns |
| IGSF5 | 42 (± 3) | 32 (± 2) | 0.003 | ** |
| ILK | 3287 (± 158) | 3627 (± 171) | <0.001 | *** |
| JAM3 | 38 (± 2) | 32 (± 2) | 0.022 | * |
| LLGL1 | 596 (± 34) | 710 (± 39) | <0.001 | *** |
| LLGL2 | 1973 (± 37) | 1894 (± 36) | 0.012 | * |
| MAGI1 | 629 (± 10) | 432 (± 13) | <0.001 | *** |
| MARK2 | 2945 (± 110) | 2613 (± 103) | <0.001 | *** |
| MARVELD2 | 3047 (± 47) | 2653 (± 37) | <0.001 | *** |
| MPDZ | 35 (± 2) | 49 (± 3) | <0.001 | *** |
| MPP5 | 3278 (± 137) | 2786 (± 116) | <0.001 | *** |
| MPP6 | 696 (± 27) | 1245 (± 34) | <0.001 | *** |
| OCLN | 1960 (± 45) | 1260 (± 25) | <0.001 | *** |
| PARD3 | 4869 (± 230) | 3752 (± 183) | <0.001 | *** |
| PARD6A | 113 (± 4) | 89 (± 5) | <0.001 | *** |
| PARD6B | 1777 (± 248) | 1054 (± 143) | <0.001 | *** |
| PECAM1 | 57 (± 5) | 50 (± 4) | 0.087 | ns |
| PRKCI | 2164 (± 90) | 1755 (± 67) | <0.001 | *** |
| PRKCZ | 1560 (± 52) | 1305 (± 39) | <0.001 | *** |
| PTEN | 3520 (± 184) | 3301 (± 169) | <0.001 | *** |
| RAC1 | 8457 (± 197) | 6278 (± 198) | <0.001 | *** |
| RDX | 13872 (± 452) | 13304 (± 463) | 0.024 | * |
| RHOA | 5676 (± 119) | 5232 (± 106) | <0.001 | *** |
| ROCK1 | 1617 (± 107) | 1431 (± 94) | <0.001 | *** |
| SMURF1 | 548 (± 25) | 439 (± 16) | <0.001 | *** |
| SPTAN1 | 4046 (± 160) | 3859 (± 150) | 0.048 | * |
| SPTB | 37 (± 1) | 31 (± 1) | 0.017 | * |
| SYMPK | 2682 (± 88) | 2880 (± 116) | 0.003 | ** |
| TIAM1 | 72 (± 5) | 83 (± 8) | 0.053 | ns |
| TJAP1 | 314 (± 10) | 321 (± 7) | 0.451 | ns |
| TJP1 | 3733 (± 235) | 3592 (± 224) | 0.035 | * |
| TJP2 | 3563 (± 131) | 3228 (± 73) | <0.001 | *** |
| TJP3 | 2905 (± 93) | 2876 (± 93) | 0.616 | ns |
| VAPA | 14435 (± 348) | 9585 (± 260) | <0.001 | *** |
| WAS | 28 (± 1) | 24 (± 1) | 0.227 | ns |
| WASL | 5332 (± 113) | 4467 (± 98) | <0.001 | *** |
| WHAMM | 1003 (± 42) | 692 (± 29) | <0.001 | *** |
| YBX3 | 4810 (± 280) | 4565 (± 224) | 0.096 | ns |
